# RET is a sex-biased regulator of intestinal tumorigenesis

**DOI:** 10.3389/fgstr.2023.1323471

**Published:** 2024-01-16

**Authors:** Sean T. Koester, Naisi Li, Neelendu Dey

**Affiliations:** ^1^Translational Science and Therapeutics Division, Fred Hutchinson Cancer Center, Seattle, WA, United States; ^2^School of Medicine, Kansas University Medical Center, Kansas City, KS, United States; ^3^Microbiome Research Initiative, Fred Hutchinson Cancer Center, Seattle, WA, United States; ^4^Department of Medicine, Division of Gastroenterology, University of Washington, Seattle, WA, United States

**Keywords:** colorectal cancer, microbiome, RET, sexual dimorphism, Apc Min/+ mice

## Abstract

*Ret* is implicated in colorectal cancer (CRC) as both a proto-oncogene and a tumor suppressor. We asked whether RET signaling regulates tumorigenesis in an *Apc*-deficient preclinical model of CRC. We observed a sex-biased phenotype: *Apc^Min^*^/+^*Ret*+/- females had significantly greater tumor burden than *Apc^Min^*^/+^*Ret*+/- males, a phenomenon not seen in *Apc^Min^*^/+^ mice, which had equal distributions by sex. Dysfunctional RET signaling was associated with gene expression changes in diverse tumor signaling pathways in tumors and normal-appearing colon. Sex-biased gene expression differences mirroring tumor phenotypes were seen in 26 genes, including the *Apc* tumor suppressor gene. *Ret* and *Tlr4* expression were significantly correlated in tumor samples from female but not male *Apc^Min^*^/+^*Ret*+/- mice. Antibiotics resulted in reduction of tumor burden, inverting the sex-biased phenotype such that microbiota-depleted *Apc^Min^*^/+^*Ret*+/- males had significantly more tumors than female littermates. Reconstitution of the microbiome rescued the sex-biased phenotype. Our findings suggest that RET represents a sexually dimorphic microbiome-mediated “switch” for regulation of tumorigenesis.

## Introduction

Colorectal cancer (CRC) is one of the most common cancers globally. *Ret*, which is critical in enteric nervous system (ENS) development and maintenance, has been implicated as both a proto-oncogene (1) and tumor suppressor (2) in CRC. While RET fusions in metastatic CRC portend a poor prognosis (3), specific RET inhibitors such as selpercatinib and pralsetinib have demonstrated efficacy in targeting oncogenic RET rearrangements [reviewed at (4, 5)]. *Apc* encodes a tumor suppressor and is commonly mutated in CRC. We assessed interactions of *Apc* and *Ret* through a crossbreeding experiment using *Apc^Min^*^/+^ mice and *Ret*+/- mice (6). We administered 1.5% dextran sodium sulfate (DSS) to induce colonic tumors in *Apc^Min^*^/+^*Ret*+/- progeny (as well as littermate controls with a mutation in *Apc* only, *Ret* only, or neither). We expected that *Apc^Min^*^/+^ mice would develop many intestinal tumors compared to wild-type or *Ret*+/- mice, which are not predisposed to developing tumors, but it was unknown whether mice harboring mutations both in *Apc* and *Ret* would develop a greater, lesser, or equivalent number of tumors compared to *Apc^Min^*^/+^ mice. We hypothesized that if tumorigenesis is regulated by RET signaling, then we should observe a change in tumor burden in *Apc^Min^*^/+^*Ret*+/- mice that is either decreased compared with *Apc^Min^*^/+^ mice if RET signaling promoted tumorigenesis or increased if RET suppressed tumorigenesis.

## Results

**Figure 1:**
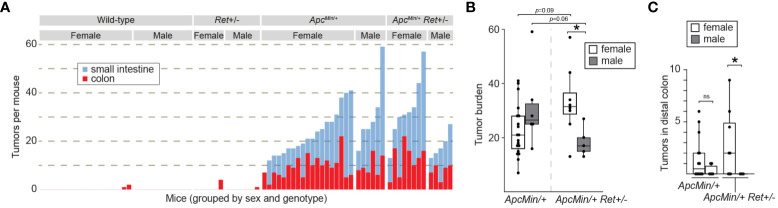
RET signaling regulates intestinal tumorigenesis in a sex-dependent manner. **(A)** Tumor burden in male and female wild-type, *Ret*+/-, *Apc^Min^*^/+^, and *Apc^Min^*^/+^*Ret*+/- mice treated with 1.5% DSS. Each stacked bar represents one mouse. **(B, C)** Total intestinal and distal colonic tumor burdens. *: p<0.05, ns: not significant.

Consistent with expectations, (i) *Apc^Min^*^/+^ mice (*n*=25) had a significantly greater tumor burden than wild-type littermates (*n*=31) (mean ± SEM: 22.6 ± 2.2 vs 0.16 ± 0.12, *p*<10^-8^ [females]; 31.2 ± 6.1 vs 0 ± 0, *p*=0.004 [males]; **Figures 1A, B**; **Supplementary Table 1**); (ii) *Ret*+/- mice (*n*=13) had tumor burdens not significantly different from wild-type littermates (*n*=31) (0.7 ± 0.7 vs 0.2 ± 0.1 [females]; 0.1 ± 0.1 vs 0 ± 0, [males]; **Figure 1A**; **Supplementary Table 1**); and (iii) compared to *Apc^Min^*^/+^ mice that did not receive DSS (*n*=30), DSS-treated *Apc^Min^*^/+^ mice developed more colonic tumors (9.3 ± 1.0 vs 1.1 ± 0.3, *p*<10^-6^ [females]; 10 ± 1.7 vs 1.8 ± 0.3, *p*=0.004 [males]). In the experimental *Apc^Min^*^/+^*Ret*+/- cohorts, there was no overall significant difference in tumor burden compared to *Apc^Min^*^/+^ mice (two-way ANOVA; **Figure 1A**). However, we observed a sex-biased interaction of *Ret* and *Apc*: total (colonic plus small intestinal) tumor burden was significantly greater in female *Apc^Min^*^/+^*Ret*+/- mice compared to male *Apc^Min^*^/+^*Ret*+/- mice (3-way ANOVA, interaction between *Apc*, *Ret*, and sex covariates: *p*<0.003, F_1,74 = _10; **Figure 1B**). Compared to males, female *Apc^Min^*^/+^*Ret*+/- mice had significantly greater tumor burden in the distal colon, which we defined as the last 25% of the colon by length (2.94 ± 1.17 vs 0 ± 0, *p*<0.04, Student’s two-tailed *t*-test; **Figure 1C**). In the small intestine, *Apc^Min^*^/+^*Ret*+/- females had more tumors than males (20.5 ± 4.2 vs 10.6 ± 2.3, *p*=0.06, Student’s two-tailed *t*-test). These sex-biased differences did not exist among *Apc^Min^*^/+^ mice.

Male *Apc^Min^*^/+^*Ret*+/- mice had longer small intestines than female *Apc^Min^*^/+^*Ret*+/- mice (36.4 ± 0.37 cm vs 33.9 ± 0.85 cm; *p*<0.03, Student’s two-tailed *t*-test). While of unclear biological significance, it excludes small intestinal length as a potential confounder for the difference in small intestinal tumor burden, which was greater in females. Otherwise, there were no sex-based differences in tumor size, intestinal lengths, or whole gut transit times (**Supplementary Table 1**). Within control cohorts, there were also no sex-based differences in these metrics.

**Figure 2:**
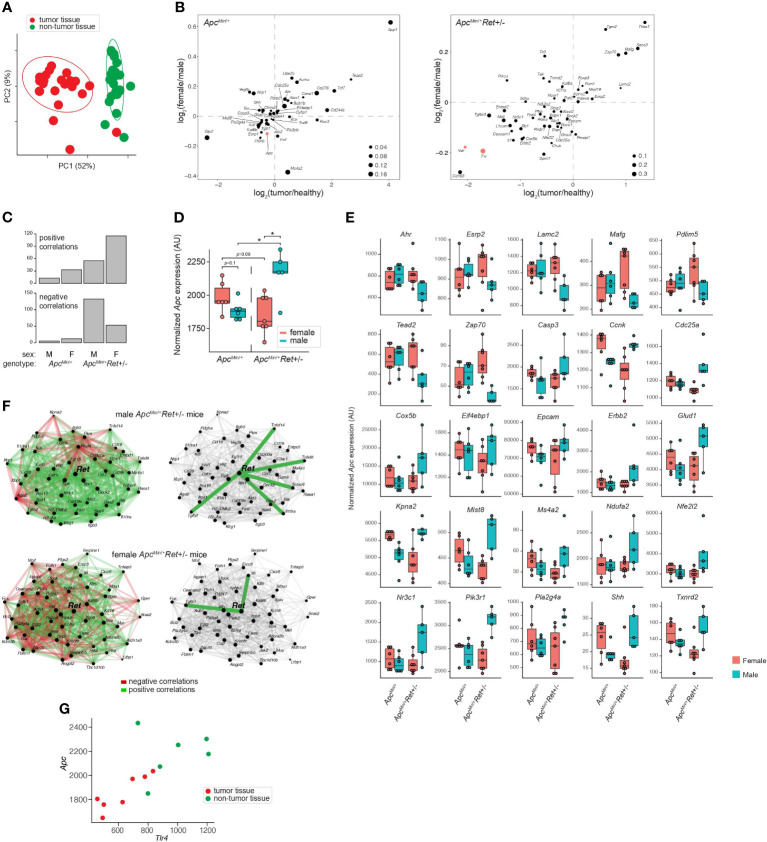
Global and gene-specific variation in colonic gene expression is regulated by RET signaling in a sex-dependent manner. **(A)** Principal coordinate analysis of global gene expression variation across samples. **(B)** Genes whose expression was significantly affected by the interaction between sex, genotype, and sample type. 2D enrichment plots of genes significantly enriched in tumors (x-axis) or in females (y-axis) in *Apc^Min^*^/+^ (left) and *Apc^Min^*^/+^*Ret*+/- (right) mice. The dot sizes represent log of fold change of expression in each genotype compared to the other. Three genes referenced in the text are colored red. **(C)** Numbers of genes that are significantly correlated with *Apc* expression in different sex and genotype contexts. **(D)** Tumor *Apc* expression split by cohort. **(E)** 25 genes, other than Apc, whose expression in tumors mirrored the sex-biased genotype-dependent tumor phenotype based on the mean expression for each group. Plots are organized by genes that first follow the tumor phenotype (U shaped) and then the inverse. **(F)** Sex biases in *Ret* gene networks in *Apc^Min^*^/+^*Ret*+/- mice. **(G)** *Tlr4* and *Apc* correlation in *Apc^Min^*^/+^*Ret+/-* females. *: p<0.05.

To define *Ret*’s role in tumor signaling pathways, we profiled gene expression in colonic tumors (*n*=24) and healthy-appearing colonic tissues (*n*=24) from male and female *Apc^Min^*^/+^ and *Apc^Min^*^/+^*Ret+/-* mice (**Supplementary Table 2A**). Global gene expression profiles clustered predominantly by whether they represented tumors or healthy tissues (*p*=0.001, PERMANOVA [permutational multivariate analysis of variance using distance matrices] using Bray-Curtis dissimilarity; **Figure 2A**). Expression levels of a subset of genes were nonetheless affected by the interaction between sex, genotype, and tissue sample type (ANOVA: *p*<0.05; **Figure 2B**). Consistent with their established biological roles, *Apc*, *Fxr* (*Wnt* pathway antagonist; bile acid receptor), and *Vdr* (vitamin D receptor; bile acid receptor) were expressed at higher levels in healthy tissues. In tumors, *Myc* (oncogene), *Lgr5* (stem cell marker), and *Wnt5a* (*Wnt* pathway activator) were expressed at higher levels and were affected by the interaction between sex and genotype (ANOVA: *p*<0.05). Consistent with *Fxr*’s role as a negative regulator of bile acid synthesis, total bile acid concentrations were higher in females than males (3,604.2 ± 418.2 ng/mg stool vs 2,410.84 ± 177.2 ng/mg stool, *p*=0.01, Student’s two-tailed *t*-test).

*Ret* deficiency appeared to dysregulate homeostatic gene expression networks, resulting in a dramatic increase in numbers of genes significantly correlated with *Apc* expression in tumor samples (**Figure 2C**). Interestingly, *Apc^Min^*^/+^*Ret*+/- female mice had more positive than negative correlations, while *Apc^Min^*^/+^*Ret*+/- male mice had more negative than positive correlations (**Figure 2C**). The genes correlated with *Apc* are implicated in diverse pathways, suggesting global effects on gene expression (**Supplementary Table 2B**). In healthy samples, *Apc* expression was greater in *Apc^Min^*^/+^*Ret*+/- males than in females (2,452 ± 58 arbitrary units [AU] vs 2,181 ± 83 AU, *p*<0.03, Student’s two-tailed *t*-test). In tumor samples, *Apc^Min^*^/+^*Ret+/-* males had significantly higher *Apc* expression than both *Apc^Min^*^/+^*Ret+/-* females (2,160 ± 80 AU vs 1,854 ± 55 AU, *p*<0.02, Student’s two-tailed *t*-test) and *Apc^Min^*^/+^ males (2,160 ± 80 AU vs 1,885 ± 29 AU, *p*=0.02, Student’s two-tailed *t*-test). Notably, within tumor samples, *Apc* expression was higher in male *Apc^Min^*^/+^*Ret+/-* mice than in female *Apc^Min^*^/+^*Ret+/-* mice, and this trend was opposite in *Apc^Min^*^/+^ mice — the inverse of the sex-biased genotype-dependent tumor phenotype (**Figure 2D**). Including *Apc*, tumor-intrinsic expression levels of 26 genes mirrored tumor phenotypes (exactly or inversely) and were significantly affected by interactions between sex and genotype (*p*<0.05, two-way ANOVA) (**Figure 2E**). While *Apc* expression levels varied with tumor phenotypes in such a manner that it may plausibly explain the underlying biology, the biological significance of the other 25 genes is more challenging to interpret and underscores complexity of gene network interactions.

Strikingly, tumor-intrinsic *Ret*-centric gene expression networks were entirely non-overlapping between male and female *Apc^Min^*^/+^*Ret+/-* mice (**Figure 2F**). In tumors harvested from *Apc^Min^*^/+^*Ret+/-* males, we observed correlations between *Ret* and *Il10ra*, *Tnfsf14*, *Tnfsf8*, *Tnfrsf4*, and *Tgfb2* (r≥0.89, *p*<0.05, Pearson correlation), which are involved in anti-inflammatory responses. In contrast, in *Apc^Min^*^/+^*Ret+/-* females, *Ret* was significantly correlated with *Tgfb1* and with *Il6* (r≥0.78, *p*<0.05, Pearson correlation), both of which are involved in upregulating the immune system. These findings suggest links between the immune system and *Ret*, whereby loss of functional RET signaling leads to a pro-inflammatory state in females and an anti-inflammatory state in males.

Intriguingly, *Ret* expression was significantly correlated with *Tlr4* expression in tumor samples from all cohorts of mice (r≥0.79, *p*<0.04, Pearson correlation) except for the male *Apc^Min^*^/+^*Ret+/-* mice. TLR4 is critical in microbial pattern recognition and is overexpressed in colorectal cancers and adenomas compared to healthy tissues in humans (7). *Tlr4* was correlated with *Apc* in *Apc^Min^*^/+^*Ret+/-* female mice (tumor samples: r=0.88, *p*<0.01; all samples: r=0.73, *p*<0.005, Pearson correlation; **Figure 2G**) but not in other cohorts.

**Figure 3:**
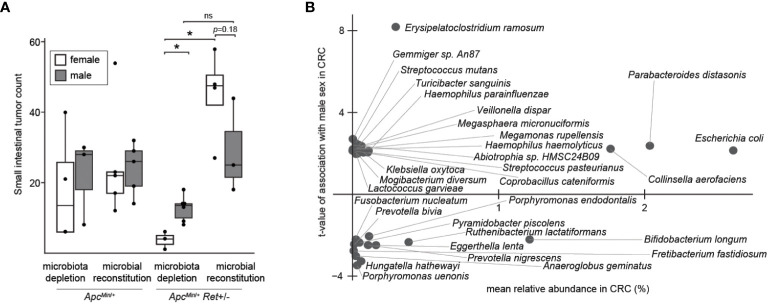
The microbiome as a potential sex-biased driver of intestinal tumorigenesis. **(A)** Effects of microbiota depletion with or without gut microbial reconstitution on tumor burden. **(B)** Bacterial species enriched in males (up) or females (down) in human CRC microbiomes, adjusting for biases in non-CRC cohorts. *: p<0.05, ns: not significant.

Thus, we next tested the role of the gut microbiome in driving sex bias in tumor phenotypes. We either administered chronic antibiotics or first depleted the native microbiota with antibiotics and then reconstituted the microbiota via oral gavage using a uniform fecal suspension. Chronic antibiotics reduced tumor burden, significantly more so in females, and resulted in complete inversion of the sex-biased tumor phenotype: microbiota-depleted *Apc^Min^*^/+^*Ret+/-* females had significantly fewer tumors than males (3.7 ± 1.5 vs 12.7 ± 1.5, *p*=0.005, two-tailed Student’s *t*-test; **Figure 3A**). The interaction between sex and microbiome status (harboring or lacking a microbiome) was significant (*p*<0.02, *F*_1,12 = _7, two-way ANOVA).

Finally, we asked whether human CRC data support our findings. Approximately 10% of CRCs are reported as harboring *Ret* mutations (2). Intriguingly, in The Cancer Genome Atlas (8), CRCs with *Ret* mutations occurred predominantly in females (z=2.98, *p*<0.003). Further, in a reanalysis of published CRC microbiome surveys (9), we found that 68 bacterial species were differentially abundant between sexes in CRC (**Figure 3B**); of these, only 11 species were also differentially abundant in the healthy cohort, evidencing CRC-specific sex biases. *E. coli* was more abundant in males with CRC than in females with CRC, whereas in the healthy cohort, *E. coli* was more abundant in females than in males. In contrast, *Fusobacterium nucleatum* was more abundant in females than in males in the CRC cohort. *F. nucleatum*, which is linked to CRC progression and metastasis, has been associated with CpG methylation and a CpG island methylator phenotype, which is associated with the female sex (10).

## Discussion

The answer to our initial question — is RET an oncogene (1) or tumor suppressor (2) in CRC? — appears to be complicated: it depends on sex and the microbiome. Underlying the phenotype that we discovered were significant differences in tumor burden in the distal colon. Interestingly, public datasets suggest that *Ret* mutations in CRC and CRC microbiome signatures are both sex-biased. Our study offers proof-of-principle that CRC risk-modulating gut microbial effects depend on sex and genetics, and they underscore the importance of evaluating sex as a biological variable in research and of reporting the sexes of both human and non-human study participants.

A limitation of this study is that the specific cellular players remain unknown. Our gene expression data suggest that both tumor-intrinsic and tumor-extrinsic cells are disrupted by *Ret* insufficiency, and that the resulting *Ret* gene networks starkly differ by sex. RET is a critical player in the ENS, which transmits intestinal growth signals via the GLP-2 pathway (11, 12), potentially facilitates CRC metastasis (13, 14), and regulates immunity (15) and physiology (16). Enteric neurons express estrogen receptor (17) so could form the basis for sex-biased microbiome-dependent tumorigenesis. Future studies should elucidate tumor cell-autonomous effects versus effects of a RET-deficient ENS. This research could lead to novel personalized CRC prevention tactics.

## Materials and methods

### Animal husbandry

Male and female *Apc^Min^*^/+^, *Ret*+/-, and *Apc^Min^*^/+^*Ret*+/- mice (C57BL/6 background) were studied using methods approved by the Institutional Animal Care and Use Committee of Fred Hutchinson Cancer Center (protocol 51049). Mice from the same litter were co-housed regardless of differences in genotype, thereby leading to shared microbiota due to coprophagy. Mice were fed *ad libitum* with PicoLab Mouse Diet 5058. DSS (1.5% by volume) was administered in drinking water for 7 days to mice at 6-8 weeks of age. Following DSS exposure, mice were given standard drinking water for the remainder of the experiment. Mice were euthanized ~6 weeks after DSS exposure (or earlier if exhibiting symptoms of high tumor burden) using aerosolized 1.5% isoflurane delivered through a precision vaporizer. At euthanasia, small intestines and colons were harvested, rinsed in ice cold phosphate buffered saline to remove fecal material, and opened longitudinally for tumor counting and characterization. Fresh fecal pellets were snap-frozen in liquid nitrogen and stored at -80°C until use. *Tumor localization analysis*. Digital images of colons were equally split into quartiles. Tumors spanning different segments of the colon had fractions of tumors counted in each segment. These fractions were estimated as 0.25, 0.5, or 0.75, and the fractions for each tumor add up to 1. *Microbiome manipulation studies*. Vancomycin (1 g/L), metronidazole (1 g/L), and neomycin (0.5 g/L) antibiotics (Millipore Sigma, St. Louis, MO) were delivered via drinking water containing 2% sucrose (20 g/L) over 10 days or, in the experiment involving chronic antibiotic administration, cyclically throughout the duration of the experiment. Recolonization was performed via oral gavage of a fecal suspension from female *Apc^Min^*^/+^*Ret+/-* mice. Due to enhanced DSS toxicity in the setting of antibiotic use, mice receiving antibiotics were not given DSS; therefore, small intestinal tumor counts were used as readouts in these experimental cohorts.

### Gut transit time measurements

Using previously published methods (18), mice were gavages with 200 μL per mouse of a sterilized 6% red carmine dye solution (Sigma C1022) and monitored for time to initial passage per rectum. Gavages were performed by the same individual (N.L.) within a consistent time frame in all mice in order to minimize variability.

### RNA isolation

After flushing with PBS, gut segments were stored in RNA*later* (Thermo Fisher Scientific Inc., Waltham, MA) at 4°C for 24 hours and then transferred to -20°C for storage until use. To isolate RNA, 20 mg of each sample was placed into a tube containing 0.1 mm zirconium beads, a 4 mm steel ball, and homogenization buffer before mechanical disruption using a TissueLyser II (Qiagen, Hilden, Germany). RNA was purified using RNeasy Mini kits (Qiagen).

### Colonic tumor and non-tumor gene expression profiling

Gene expression data were generated using NanoString nCounter^®^ Tumor Signaling 360 Panels in conjunction with a 55-gene Panel Plus.

### Analysis of published human microbiome datasets

We utilized *curatedMetagenomicData*, an R package linked to a database of curated data from nearly 100 microbiome surveys (9). We filtered in stool samples from individuals with CRC and healthy individuals at least 18 years of age, none of whom reported current antibiotic use. We identified 625 stool samples from individuals with CRC (392 males and 233 females) and 5,221 stool samples from otherwise healthy individuals (2,178 males and 3,043 females). To control for differences in age and study population, we included only the subset of individuals from the healthy cohort who were (i) from the same countries represented in the CRC cohort and (ii) within 2 years of age of an individual in the CRC cohort. Ultimately, this resulted in a healthy cohort comprised of 1,707 individuals (911 males and 796 females). We then used *corncob*, an R package that uses beta-binomial regression to model relative abundances and identify bacteria significantly associated with covariates of interest (19), to identify bacterial species that were significantly differentially abundant in CRC between sexes.

### Data analysis

Statistical comparisons were performed in R (version 4.0.0). Figures were generated using R using native functions as well as the *ggplot2* (version 3.1.0) and *pheatmap* (version 1.0.12) packages. Figures were assembled in Adobe Illustrator.

## Data availability statement

The previously published sequencing datasets re-analyzed in this study can be found in the online curated Metagenomic Data database, which lists the names of the original repositories along with corresponding accession numbers. The gene expression data we generated and present here are reported in **Supplementary Table 2**.

## Ethics statement

The animal study was approved by the Institutional Animal Care and Use Committee of Fred Hutchinson Cancer Center. The study was conducted in accordance with the local legislation and institutional requirements.

## Author contributions

SK: Conceptualization, Data curation, Formal analysis, Investigation, Methodology, Software, Validation, Visualization, Writing – original draft, Writing – review & editing. NL: Conceptualization, Data curation, Formal analysis, Investigation, Methodology, Validation, Writing – original draft, Writing – review & editing. ND: Conceptualization, Data curation, Formal analysis, Funding acquisition, Investigation, Methodology, Project administration, Resources, Software, Supervision, Validation, Visualization, Writing – original draft, Writing – review & editing.

## Supplementary material

Supplementary Table 1 Individual mouse data. Unique mouse identification numbers, cage identification numbers, sex, genotype, age at time of euthanasia, microbiota, whether DSS was used, diet, transit times, small intestinal length, cecal length, colonic length, and tumor counts by location.

Supplementary Table 2 Gene expression data. **(A)** Normalized data from the NanoString nCounter^®^ Tumor Signaling 360 Panels in conjunction with a 55-gene Panel Plus**. (B)** Genes significantly correlated with *Apc* expression in *Apc^Min^*^/+^ and *Apc^Min^*^/+^*Ret*+/- mice.
